# Granulomatosis With Polyangiitis Presenting as Complete Hearing Loss

**DOI:** 10.7759/cureus.24711

**Published:** 2022-05-03

**Authors:** Brandon H Busch, David Wilhelm, Paul Johnson, Mark Pfeifer

**Affiliations:** 1 Internal Medicine, University of Louisville, Louisville, USA

**Keywords:** canca, proteinase 3 (pr3)-positive granulomatosis with polyangiitis (gpa), diagnostic delay, autoimmune vasculitis, mixed hearing loss, anca associated vasculitis, wegener’s granulomatosis, granulomatosis with polyangiitis (gpa)

## Abstract

Granulomatosis with Polyangiitis is a rare autoimmune vasculitis that is classically characterized by effects on the upper respiratory tract, lungs, and kidneys. Delay in diagnosis is often attributed to variable and sequential presentation of symptoms rather than concurrent symptomatology. It is important to recognize the wide range of initial presenting symptoms as early diagnosis and treatment is critical in preventing potentially irreversible damage resulting from delayed diagnosis. We present a case of a 29-year-old male with history of mixed sensorineural-conductive hearing loss presumed to be secondary to chronic otitis media who presented to the emergency department with complaint of hematemesis with a subsequent diagnosis of granulomatosis with polyangiitis.

## Introduction

Granulomatosis with polyangiitis (GPA) is a rare autoimmune condition, with an incidence of 20 per million population per year in Europe and North America, that is classically characterized by effects on the upper respiratory tract, lungs, and kidneys [1]. These effects are a result of small-to-medium vessel vasculitis, causing granuloma formation and tissue necrosis. The initial presenting symptom of GPA is variable, which makes diagnosis a particular challenge. Rodriques et al. have shown that the most commonly affected organs/systems at presentation were the upper airways (64%) followed by the lungs (34%), kidneys (25%), eyes (25%), musculoskeletal system (25%), skin (11%), and nervous system (7%) [2]. However, ENT involvement has also been implicated in GPA. Delay in diagnosis is a significant challenge in these patients with vasculitis, especially given its low incidence in the general population. This delay in diagnosis can result in irreversible morbidity and mortality.

## Case presentation

The patient was a 29-year-old male who presented to the emergency department with an initial complaint of hematemesis. He reportedly just finished eating dinner with his family and started to “vomit” large amounts of blood. His medical history was significant for a diagnosis of recurrent otitis media over the past year, for which he had tympanostomy tubes placed, as well as a recent diagnosis of mixed sensorineural-conductive hearing loss. He had no significant family history. He was a nonsmoker, never used drugs, and worked as an industrial engineer without any apparent environmental exposure.

Further questioning of possible symptoms from the past year elicited additional pertinent information. Approximately seven months prior to admission he began experiencing sinus congestion issues that he attributed to frequent mask-wearing due to the severe acute respiratory syndrome coronavirus 2 (SARS-CoV-2) pandemic. He went to an immediate care clinic multiple times and completed several courses of antibiotics without relief. At the last visit to an immediate care clinic, he was given a course of steroids with some improvement in symptoms. Two months prior to admission, he started having nose bleeds that would last up to 10 minutes. The nose bleeds would occur with sneezing or spontaneously.

Five months prior to admission he began having a feeling of congestion in his left ear followed by subjective hearing loss. An otolaryngology provider diagnosed him with chronic otitis media. Tympanostomy tube placement was attempted in the clinic but was prevented by granulation tissue in the ear canal per the surgeon. The otolaryngologist accomplished surgical placement under anesthesia, but there was no improvement in congestion or hearing loss. Audiology evaluation was pursued, and he was diagnosed with mixed conductive-sensorineural hearing loss without a clear etiology. Two months prior to admission the patient began experiencing intermittent hemoptysis and gross hematuria.

**Figure 1:**
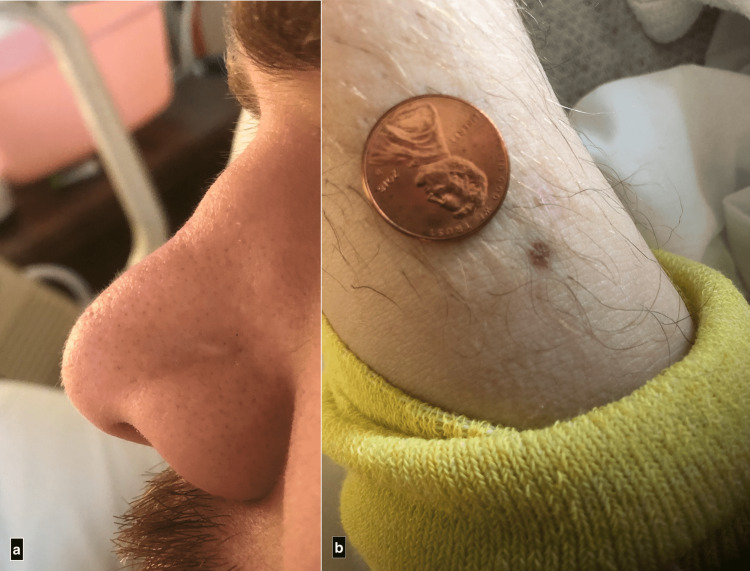
(a) Saddle-Nose Deformity; (b) Petechiae a. The patient’s family noted changes in his facial features in the year leading up to admission, most notable being the structure of his nose. b. Petechiae were present on the patient’s thigh and bilateral ankles. The lesions were non-blanching, non-palpable, and ranged from 1-3 mm in size.

On the day of admission, he had complete hearing loss bilaterally requiring the use of a voice-to-text phone application to communicate. Saddle nose deformity was present (Figure 1a). Rhonchi were appreciated in bilateral lung fields without increased work of breathing. The skin exam was notable for non-palpable, non-blanching petechial lesions 2 mm in diameter on bilateral ankles and 3 mm petechial lesions on the right thigh (Figure 1b).

**Figure 2:**
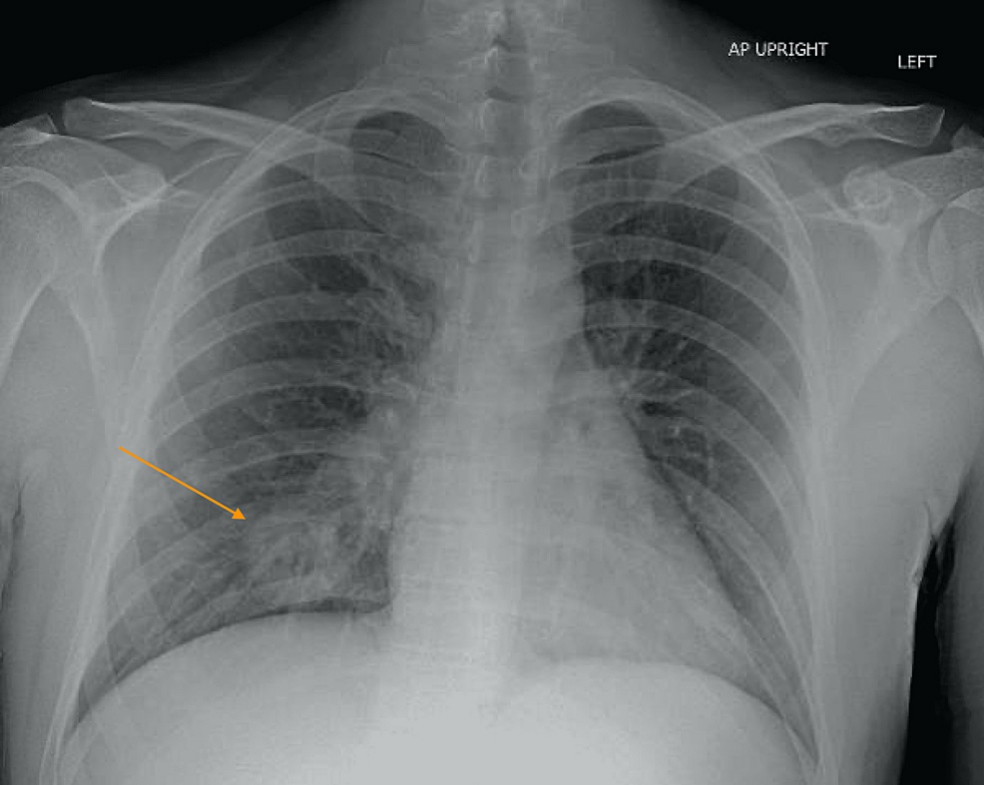
Chest x-ray showing the presence of a mass-like opacity in the medial right lung base measuring 4.1 x 4.0 cm with potential cavitation, as indicated by the arrow.

**Figure 3:**
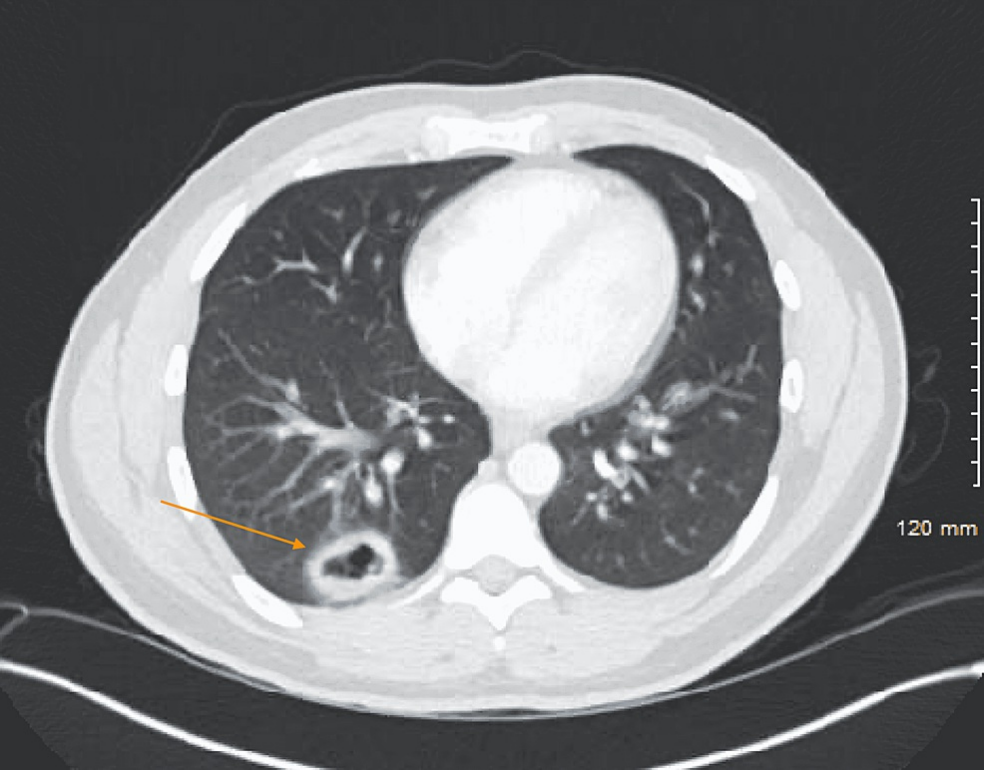
CT scan of the chest revealing the presence of 2.9 x 3.7 cm posterior right lower lobe cavitary nodule (as indicated by the arrow) as well as multiple smaller bilateral pulmonary nodules with shaggy indistinct margins.

Electrolytes were normal. WBC was 9.5 with neutrophilic predominance. C-reactive protein (CRP) was 137 and erythrocyte sedimentation rate (ESR) 104. Urinalysis was notable for 50+ RBC, 30-49 WBC without bacteria. Kidney function was normal. Acid-fast bacilli and sputum cultures were negative. Renal ultrasound and echocardiogram were both normal. Chest x-ray showed a mass-like opacity in the medial right lung base measuring 4.1 x 4.0 cm with potential cavitation (Figure 2). CT scan showed a 2.9 x 3.7 cm posterior right lower lobe cavitary nodule as well as multiple smaller bilateral pulmonary nodules with shaggy indistinct margins (Figure 3). Antineutrophil Cytoplasmic Autoantibody, Cytoplasmic (C-ANCA) was later found to be positive with a titer of 1:40 and proteinase 3 (PR3) antibodies were elevated at 3.2 (Table 1).

**Table 1:** Biochemical testing BUN: Blood urea nitrogen; AST: Aspartate Transaminase; ALT: Alanine Transaminase; ALP = Alkaline Phosphatase; WBC: White Blood Cells; Hgb: Hemoglobin; PT: Prothrombin Time; PTT: Partial Thromboplastin Time; INR: International Normalized Ratio; RBC: Red Blood Cell; LE: Leukocyte Esterase; Urine SC: Urine Squamous Cells; CRP: C-Reactive Protein; ESR: Erythrocyte Sedimentation Rate; Comp: Complement; GBM: Glomerular Basement Membrane; ANA: Antinuclear Antibody; C-ANCA: Cytoplasmic-Antineutrophilic Cytoplasmic Antibody; PR3: Proteinase-3 Unit abbreviations: mmol = millimole; L: liter; mg: milligram; dL: deciliter; microL: microliter; HPF: high power field; microg: microgram; Hr: hour; AI: antibody index

Test	Result	Normal Range	Test	Result	Normal Range
Sodium	135 mmol/L	135-143	PT	12.0 seconds	9.7-11.5
Potassium	4.0 mmol/L	3.7-5.0	PTT	21.6 seconds	23.3-33.2
Chloride	97 mmol/L	100-108	INR	1.1	0.8-1.1
Bicarbonate	28 mmol/L	22-30	Urine Blood	Large	Negative
BUN	8 mg/dL	6-20	Urine RBC	50+/HPF	None
Creatinine	0.94 mg/dL	0.64-1.27	Urine WBC	30-49/HPF	None
Glucose	131 mg/dL	70-110	Urine LE	Large	Negative
Calcium	8.6 mg/dL	8.4-10.2	Urine Nitrite	Negative	Negative
Protein	7.2 gram/dL	6.3-8.2	Urine Bacteria	None	None
Albumin	2.5 gram/dL	3.6-4.7	Urine SC	None	None
AST	32 units/L	12-38	D-Dimer	2.94 microg/mL	0.19-0.74
ALT	24 units/L	<32	CRP	137 mg/L	<10
ALP	78 units/L	34-106	ESR	104 mm/Hr	<15
Total Bilirubin	0.8 mg/dL	0.4-1.3	Comp C3	188 mg/dL	80-150
WBC	9.5 x 10^3^/microL	4.1-10.8	Comp C4	66 mg/dL	18-55
Hgb	9.9 gram/dL	13.7-17.5	GBM-antibody	<1.0 AI	<1.0 AI
Platelets	344 x 10^3^/microL	140-370	ANA	Negative	Negative
Neutrophil %	78.4%	34.0-69.5	C-ANCA titer	1:40 AI	<1:20
Neutrophil #	7.5 x 10^3^/microL	1.7-6.0	PR3 Antibody	3.2 AI	Negative

**Figure 4:**
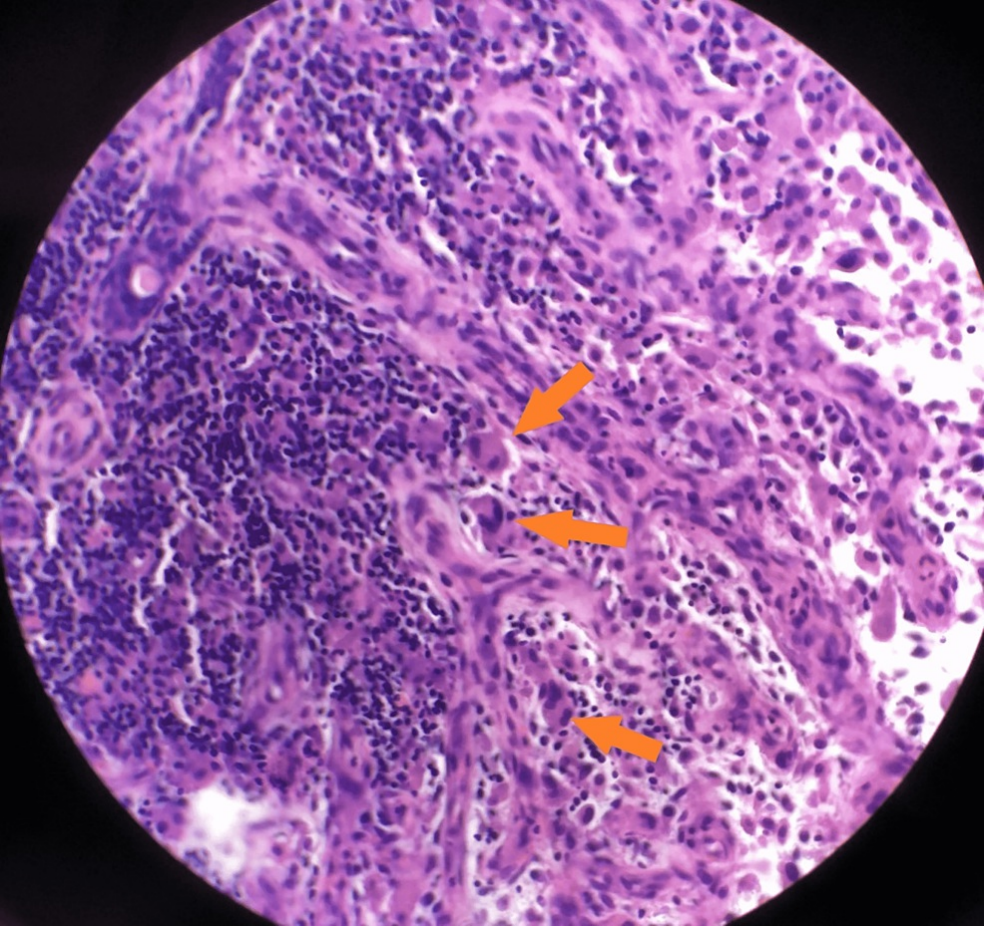
Lung biopsy: H&E stain showing the presence of multinucleated giant cells (as indicated by arrows), a nonspecific finding seen in chronic inflammation. H&E: Hematoxylin and Eosin Stain

Lung biopsy revealed marked acute and chronic inflammation including perivascular neutrophilic inflammation as well as scattered multinucleated giant cells (Figure 4). The kidney biopsy was mostly normal with only one small crescent identified on immunofluorescence.

It was presumed early that the hematemesis the patient presented with was actually the result of epistaxis or hemoptysis, given his history and initial workup. Clinical history alone for this patient placed GPA at the top of our differential. Other vasculitis syndromes were also considered including microscopic polyangiitis (MPA), eosinophilic granulomatosis with polyangiitis (EGPA), and anti-glomerular basement membrane (anti-GBM) disease (formerly Goodpasture’s). MPA presents similar to GPA with renal and lung involvement but typically lacks the upper respiratory tract symptoms such as sinusitis. EGPA will have upper respiratory symptoms such as sinusitis and lung involvement but typically manifests as more of an asthma-like history and often lacks kidney involvement. Our patient also had a normal eosinophil count, which is usually elevated in EGPA [1]. Anti-GBM will classically present with hemoptysis and hematuria but typically won’t have the upper respiratory symptoms or middle ear manifestation that we observed in our patient [3].

A diagnosis of GPA was confirmed. He was discharged with steroids as well as a close rheumatology follow-up with plans for rituximab initiation. His disease continued to progress and two months after the initial diagnosis he required supplemental oxygen at home. He has undergone multiple therapeutic bronchoscopies in order to dilate his bronchi with the goal of increasing his quality of life. His hearing has still not recovered but there is optimism that he will gain some recovery with continued immunosuppressive therapy.

## Discussion

The delay in diagnosis that this patient experienced likely resulted in irreversible injury. It is critically important that we include these rarer conditions in the differential. This patient was mislabeled as having hearing loss related to chronic otitis media. While chronic otitis media is a possible cause, a thorough history should have been pursued to rule out other possible etiology, especially when there was no improvement on antibiotics.

In one study looking at GPA and microscopic polyangiitis cases, ENT involvement was the first presenting symptom in a majority of the patients [4]. In that same study, the average duration between ENT symptoms and diagnosis of vasculitis was 14.5 months. In a retrospective study looking at all GPA diagnoses in a 20-year span in Italy, Felicetti et al. found ENT involvement in 72% of patients with otologic involvement being observed in 35% of those cases [5]. In another study of 230 GPA patients, over 95% of them showed ENT involvement with 23% being diagnosed with some degree of hearing loss [6].

The initial presenting symptom for GPA is variable and nonsequential, which makes diagnosis a particular challenge. As previously mentioned, many studies have shown that ENT involvement is often the first presenting symptom and that there is a significant delay in the diagnosis of GPA in these cases. Given the frequency of otologic manifestations of GPA, this diagnosis should be included in the differential of adults with unexplained mixed hearing loss, new-onset serous effusion, or acute otitis media in the absence of a previous history of eustachian tube dysfunction [7,8].

## Conclusions

This patient’s initial symptoms, which primarily manifested as ENT involvement and developed into complete hearing loss, began seven months prior to diagnosis. Early diagnosis may preserve hearing in patients with GPA. Therefore, it is important for clinicians to recognize the wide range of presentations in which GPA can manifest. Early recognition can help expedite diagnosis and treatment while preventing irreversible morbidity and mortality.
